# Heidegger’s philosophical foundations and his contribution to palliative nursing and spiritual care*

**DOI:** 10.1590/1980-220X-REEUSP-2024-0155en

**Published:** 2024-12-02

**Authors:** Paula Jaman-Mewes, Vera Lúcia Mendes de Paula Pessoa, Lorena Campos de Souza, Marina de Góes Salvetti

**Affiliations:** 1 Universidad de los Andes, Santiago, Chile.; 2 Universidade de São Paulo, Escola de Enfermagem, São Paulo, SP, Brazil.; 3 Universidade Estadual do Ceará, Fortaleza, CE, Brazil.

**Keywords:** Nursing, Nursing Care, Palliative Care, Philosophy, Spirituality, Enfermería, Atención de Enfermería, Cuidados Paliativos, Filosofía, Espiritualidad

## Abstract

This study aimed to reflect on the spiritual care of nursing in palliative care from the conceptual perspective of Martin Heidegger. The approximation between Heidegger’s philosophical aspects and palliative nursing practice, with a clinical view of spiritual care, promotes reflections on human existence and finitude. Being in palliative care can generate anguish, loss of meaning of life and connection with the world. The relationship of care between nurse and patient, which is established through language, favors comprehensive, intentional and humanized care, and is revealed as this relationship of care deepens. In this relational process, bonds begin to be established between nurse and patient, which can lead to authentic spiritual care. Approaching health from the spiritual dimension is a challenge, as it forces healthcare professionals to come into contact with existential issues of patients, families and themselves. Thus, reflection based on Heidegger’s philosophical aspects allows us to become aware of the facticity of death.

## RESUMEN

El objetivo de este estudio fue reflexionar sobre el cuidado espiritual de la enfermería en cuidados paliativos desde la perspectiva conceptual de Martin Heidegger. El acercamiento entre los aspectos filosóficos de Heidegger y la práctica de la enfermería paliativa, con una perspectiva clínica del cuidado espiritual, promueve reflexiones sobre la existencia y la finitud humana. Estar en cuidados paliativos puede generar angustia, pérdida de sentido de la vida y de conexión con el mundo. La relación de cuidado entre enfermera y paciente, que se establece a través del lenguaje, favorece el cuidado integral, intencional y humanizado, y se revela a medida que esa relación de cuidado se profundiza. En este proceso relacional se comienzan a establecer vínculos entre enfermera y paciente, que pueden conducir a un auténtico cuidado espiritual. Abordar la salud desde la dimensión espiritual es un desafío, pues obliga al profesional de la salud a entrar en contacto con cuestiones existenciales del paciente, de la familia y de sí mismo. Así, la reflexión basada en los aspectos filosóficos de Heidegger permite tomar conciencia de la facticidad de la muerte.

## INTRODUCTION

The current demographic change, expressed in increased life expectancy, is a global phenomenon that has been altering the epidemiological profile in different contexts, with a progressive growth in chronic and degenerative diseases, such as cancer, heart failure, chronic obstructive pulmonary disease, dementia, among others^(1)^. Children, adolescents and young adults also face life-threatening illnesses, and like older people, they experience physical, psychological, social and spiritual problems, which affect not only those who experience them, but everyone around them^(2,3)^. Tackling these diseases poses challenging questions for health systems, healthcare professionals and families, who experience the contrasts between technological advances and existential issues related to finitude, dignity and the quality of death^(4)^.

In this scenario, the importance of an efficient health system is clear, capable of expanding palliative care supply and preparing healthcare professionals to deal with the most subtle care demands, which go far beyond the physical aspect.

The World Health Organization (WHO) defines palliative care as an approach that improves the quality of life of people facing problems associated with life-threatening illnesses and their families. This approach seeks to prevent and alleviate distress through early identification, accurate assessment and treatment of pain and other problems, whether physical, psychosocial or spiritual^(3,5)^. Although palliative care may be intensified in the final phase of life, it must be offered from the moment of diagnosis, providing comprehensive support and monitoring to the person and their family throughout the entire disease process^(6)^.

Studies point to barriers in referring patients to palliative care, which can cause delays in care or even prevent access to such care. In more extreme cases, referral is delayed until a few days before death or, in many cases, does not occur at all, due to a lack of qualified personnel, high patient demand, scarcity of resources and lack of time. There is often fear and lack of knowledge about the concept and implications of palliative care, which makes it difficult to adequately meet patients’ needs^(7)^.

It is necessary to initiate palliative care early in the disease treatment^(8)^ and to address all dimensions of care, including body, mind and spirit, through authentic care that transcends the technological and curative approach^(9,10,11)^. This should also occur throughout the death and post-mortem process, considering the care offered during the mourning period^(8)^.

It is worth emphasizing that often, in the context of palliative and end-of-life care, patients and family members have difficulty making sense of what they are experiencing, and many experience hopelessness, loneliness, fear of death, guilt or frustration due to the inability to perform activities of daily living. In addition to this, temporality appears before the conscience, and the days and minutes of life are limited, generating distress, anguish and spiritual distress^(12)^. Distress can be understood from two perspectives: on the one hand, as a threat; and on the other hand, distress can occur when individuals feel that they do not have the necessary resources to face this threat^(12,13)^. Furthermore, distress may begin with anguish at the possibility that, if the symptom persists, patients will feel overwhelmed or may lose control^(13)^.

Anguish is a profound experience that emerges from cop with essential aspects of human existence, revealing the complexity of our search for meaning and purpose in a finite and uncertain world. In existentialist philosophy, anguish (in German, *angst*) is a central concept that relates to freedom, responsibility, death and the search for meaning^(14,15)^. According to Heidegger, anxiety differs from fear in that it does not have a specific object; on the other hand, it is a response to the perception of our own finitude and the lack of an absolute foundation in the world^(14)^. The context in which Heidegger describes anxiety is considered existential, due to its connection with the human condition, finitude and authenticity. Anxiety in the face of death is not simply a fear of dying; it is a deep understanding that death is an inevitable part of our existence, which allows us to live more authentically^(14)^.

Spiritual distress can arise when people cannot find transcendent meaning in their experiences or when their worldview and place in it are profoundly challenged^(16,17)^. It can also arise when a person is not in harmony with their deepest beliefs and values. This can manifest as an inner emptiness, a feeling of disconnection, or a crisis of faith^(18)^. Life-threatening illness affects the life of patients and their family, generating anguish and feelings of vulnerability, fear and uncertainty, recognizing the need for support to face their existential condition^(19)^.

Nursing care includes the spiritual dimension of human beings, which become even more relevant when facing a disease that threatens the being’s existence. Spirituality is the way people seek and experience meaning, purpose and purpose in life. It includes a sense of connection and transcendence, expressed through values and beliefs^(20)^. According to the National Cancer Institute of the United States, spirituality is present in all cultures, relating to deep feelings and beliefs, often religious, which include a sense of peace^(21)^. Many professionals, however, report that they do not feel able to provide spiritual care to patients and their families^(22)^.

Spiritual care is “care that recognizes and responds to the human spirit’s needs when confronted with trauma, illness, or grief. It includes the need for meaning and self-worth, the need to express oneself, the need to rely on faith, or simply the need to have a sensitive listener”^(23)^.

Other authors^(20)^ state that spiritual care is the recognition and attention offered to people in their search for meaning, purpose, and transcendence, in times of illness, distress, or crisis. This care focuses on recognizing and attending to the deep connections that a person has with themselves, their family, the community, nature, and the sacred or meaningful in their life. Spiritual care favors and respects patients’ beliefs, values, traditions, and spiritual practices, helping them find peace and comprehensive well-being at critical moments in their lives.

From an international perspective, strategies and interventions to facilitate spiritual care in palliative care were identified, applied by different healthcare professionals, including nurses, such as conversations between patients and a team members, interventions of religious practices, therapeutic presence, guided music therapy, multidisciplinary interventions, guided meditation, art therapy and combined interventions with multiple components, such as music, art, integrative therapy and reflection^(24)^. Early spiritual assessment, through a “spiritual history” as part of the comprehensive clinical history at the beginning of treatment, can be a good initial strategy, as it helps to identify possible spiritual needs. Examples of structured instruments for assessing spiritual history are the FICA Spiritual Assessment Tool (F: Faith or Beliefs; I: Importance or Influence; C: Community; A: Assistance) and the GES Questionnaire (Group Spirituality SECPAL)^(24)^.

But how can we address the spiritual issues of human beings? How can we face the spiritual and existential distress that arises when a person faces an advanced, incurable and irreversible disease? There are many spiritual concerns^(25)^, such as the loss of meaning of life, the feeling of guilt for wanting to hasten death, hopelessness, fear of dying, the uncertainty of finitude, the feeling of loneliness and disconnection with oneself, with others, with the sacred and with the environment^(26)^. All of these spiritual issues are aspects that technology and medicines are still unable to resolve.

There is some question about whether there is a machine or medication to treat hopelessness. Often, in the absence of an immediate and explicit solution, it is preferable to avoid discussing these spiritual issues with patients. However, it is a reality that cannot be ignored. Healthcare professionals must seek ways to promote spiritual well-being, alleviate distress and facilitate a peaceful death.

Philosopher and writer Martin Heidegger articulated phenomenology and hermeneutics, approaches that can help in understanding the existential questions of humanity. His work, “Being and Time”^(14)^, is one of the most influential of the 20^th^ century, and reveals philosophical foundations that can encourage reflection and dialogue among healthcare professionals in different contexts.

For Heidegger, a person is a “being-there”. The term “being-there”^(14,15)^ is a central concept in Heidegger’s philosophy that refers to the human being as an entity that is in the world, immersed in its concrete existential context in time and space. From this perspective, a human being is not simply an object present in the world like any other physical entity, but has a unique and singular existence. The “being-there” faces and actively engages with fundamental questions of its existence, such as the understanding of its own finitude, the search for meaning and authenticity in life, and the coping with the possibility of death^(14)^. Another Heideggerian concept is “being-in-the-world” (in German, *in-der-Welt-sein*), which refers to the way in which human beings exist and relate to the world around them. This term highlights that our existence is always immersed in a specific social and cultural context. We are not isolated entities, but our identity and understanding of being develops in relation to the world we inhabit^(14)^.

Being able to care for the “being-there” in its entirety, in the context of palliative care, is a challenge for many professionals, as it confronts them not only with the finitude of the being-there-patient’s life, but also with reflections on their own finitude. Palliative nursing professionals need to be better prepared to deal daily with human fragilities in relation to life and death, as they also experience situations of distress, anguish, fear and anxiety^(27)^. Care requires considering the patient-family-professional triad, and this act requires a space for reflection on personal and ethical values as well as on the dying process^(27,28)^.

This article aimed to reflect on spiritual care in nursing in palliative care from Martin Heidegger’s philosophical-conceptual perspective.

## CONCEPTUAL ASPECTS OF THE PHILOSOPHY OF BEING IN HEIDEGGER

Martin Heidegger was one of the most important thinkers of the 20^th^ century. He lived in Germany between 1889 and 1976. His writings continue to be studied to this day and serve as the basis for philosophical research in various fields of knowledge, including health. The foundation of Heidegger’s thought is the philosophy of being, which explores the meaning and nature of human beings in relation to their concrete existence in the world. Existential ontology, as Heidegger called it, refers to the study of how human beings live, relate to and experience their own unique and concrete existence. Reflection on being, understood as the fundamental essence of human beings, constitutes the starting point of his philosophy^(14)^.

His theological training was the beginning of his academic formation. He had a solid religious background, carried out humanistic studies and, in 1909, began his ecclesiastical career. However, after ten years, he retired and, in 1920, he was an assistant to Edmund Husserl at the University of Freiburg. His philosophical roots are inspired by Heraclitus, Aristotle, Plato and Kant^(29)^. Understanding his intellectual formation allows us to recognize the way the philosopher saw the world.

Throughout his studies, especially in his book “Being and Time”^(14)^, Heidegger developed some concepts that can contribute to reflection on humans being who experiences palliative care, focusing on the spiritual dimension. The philosopher’s theoretical framework is quite vast, but for an adequate reflective movement, we chose to establish concepts such as being-there, being-in-the-world, being-in-the-world with, existence (authentic and inauthentic), anguish, being-for-death and care, as described in Chart 1.

**Chart 1:** Essential concepts of Heidegger’s thought – São Paulo, SP, Brazil, 2024.

Concepts	Definition of the concept
Dasein there-being	Heidegger conceives of human beings as a being that exists in the world, an existential being thrown into the world. This determination points to an ontological structure^(14)^. Dasein is conceived in advance as something that is there, and is a possible being (p.147). The essential being-power of Dasein concerns the ways of dealing with the “world”, the concern for others and the being-power in relation to itself^(14,15)^.
Being-in-the-world	It contains within itself the relation of existence to being in its totality. Depending on the moment, this concept has different aspects: the analysis in relation to the world in its worldliness, being-in-the-world as co-being and being-itself, and being-in, as such^(14,15)^. The world has an existential meaning, in which different possibilities occur again: world can mean the “public” world of us or the “own” and closest surrounding (domestic) world^(14)^.
Being-in-the-world “with”	The world of Dasein is a world in common. To be within is to be with others^(14)^.
Authentic existence	It is the being that adequately assumes its existence, taking responsibility for all the ways of perceiving its presence in time^(29)^. The authentic existing being is the being that decides for itself, does not allow itself to be influenced by other that it will die, accepts death and this does not prevent it from living, does not become distressed thinking about death. The authentic being-there allows the being to live with greater joy^(30)^.
Inauthentic existence	It is the being that still denies that they will die. Then they overload themselves with things to suffocate the idea of death within themselves^(29)^. The inauthentic being ignores death, and ignorance generates anguish. The neurotic denial of death manifests itself in irritability and other illnesses^(30)^.
Anguish	It is an affective state that allows the being to open up to a part of reality. According to Heidegger, anguish generates an openness to the world as such, and this reality goes beyond being-there. It is a complex human experience in which all the systems in which life found meaning collapse. Something that makes the world be perceived as meaningless. In anguish, the being realizes that it has no defined destiny, has nowhere to project itself, and becomes aware that it is a free being. Freedom appears and the being can choose how to develop^(14,15)^. Freedom comes with responsibility, because each being realizes that actions have consequences. According to Heidegger, anxiety connects man with their being-towards death. The phenomenon of anxiety is different from fear, because it emerges from Dasein itself. The threatening element is not found anywhere, we do not know what it is that causes us anxiety, fear, on the other hand, comes from the intra-worldly^(14,15)^.
Being-towards-death	Within all the possibilities of being-there, Heidegger analyzes the possibility of death for the human being. And each being-there, individual and unrepeatable, asks about being and it is the being-there that dies. Dasein knows that death is within its possibilities, and this is an individual and non-transferable phenomenon^(14)^.
Care (*sorge*)	Care is the existential meaning of Dasein of being-there^(14,15)^. For Heidegger, care (*sorge*) can be understood as occupation (*besorgen*) and as concern (*fürsorge*), being intrinsic to being and essentially indivisible^(14)^. Care as an occupation defines a specific use that the being-there makes of those other beings that come to meet them for their use, giving them a characteristic of tools or of concern, made explicit in the relationship or way of being of a presence towards the other, i.e., a kind of relationship of intersubjectivity. Care as concern (*fürsorge*) reveals itself in being-with-the-other^(14,15)^.

### Heideggerian Phenomenology and its Contribution to Palliative Nursing

To understand the being of man, Heidegger uses the phenomenological method, which enables a journey from being-there^(14)^. Such a method reestablishes contact with the deepest human concerns.

Heidegger (1927) pointed out that the word “phenomenology” consists of two parts: “phenomenon” and “logos”. Both come from Greek terms. The concept phenomenon refers to “that which shows itself in itself”, “that which is patent”, “that which appears to consciousness”, and the concept logos refers to “speech”, i.e., to “word”. Heidegger also maintains that the being of humans is based on language and that it becomes present through dialogue^(31)^. As human beings, we express and understand the world by putting our experiences into words^(32)^. In this regard, phenomenology refers to “making seen what is shown by itself”. This is the formal meaning of phenomenological research^(14)^.

When we perceive something, we make use of our previous knowledge, experiences and positions, and from there, we begin to understand the new movement and interpret the phenomenon. This fact leads us to the importance of understanding the totality of being, which needs to be understood without fragmentation, i.e., the being-there in its totality^(33)^.

Heideggerian phenomenology offers us the possibility of discovering hidden phenomena, such as the phenomenon of being a persona in palliative care, because it allows us to be in touch with those who experience this, and, based on their discourses, we can understand and interpret what is underlying: the phenomenon itself^(34)^.

Palliative nursing cares for people who are facing illnesses that affect their entire being in the biopsychosocial and spiritual spheres. One challenge for palliative care nursing is to understand the spiritual dimension, which encompasses philosophical ideas about life, its meaning and purpose. People receiving palliative care may experience spiritual symptoms, such as anguish, unhappiness, disbelief, hopelessness, among others, which also significantly deteriorate their health^(35)^.

This new circumstance in the life of individuals and their family requires comprehensive care from the healthcare team, especially the nursing team, who are the ones who spend most of the time with patients. From a Heideggerian perspective, the act of understanding and caring constitutes an essential quality of being present^(36-37)^. Understanding requires knowing the other person’s condition as well as having the ability to feel the other person’s feelings^(36)^.

Understanding patients’ life experiences can help to qualify care. The phenomenological method, in palliative nursing, suggests going to the things themselves, manifesting in the commitment to the information shared by patients and family members^(36)^. Nurses have the possibility of investigating and revealing the health situation of a person who is undergoing palliative care, understanding the lived experience not only of their being-there, but of their being-there-in-the-world.

Caring for others involves the genuine intention of understanding the other person^(35)^. Understanding lived experience through the phenomenological method provides the foundations for qualifying personalized nursing care. In palliative nursing, the application of Heidegger’s concepts, such as “being-towards-death”, authenticity and understanding of finitude, can be observed in different studies and in practice, covering patients^(38)^, family members^(38,39)^, nurses^(27,40)^ and other healthcare professionals^(41)^.

A qualitative study that investigated the lived experience of patients and nurses in the context of palliative care highlighted that careful interpretation of patients’ concerns about death by nurses not only improves communication and understanding, but also facilitates a deep existential dialogue that reflects Heideggerian concepts, such as being-towards-death, authenticity and the reassessment of values in the care of patients at the end of life^(42)^. Another study in nursing that used Heideggerian phenomenology demonstrated the importance of assuming one’s own existence, with awareness of its finitude and the need to find meaning and value in life, even in the most advanced stages of life^(43)^.

## CONCEPT OF SPIRITUALITY AND “BEING-THERE” IN PALLIATIVE CARE

Spirituality is defined as “an intrinsic and dynamic aspect of humanity through which people seek ultimate meaning, purpose, and transcendence, and experience a relationship with themselves, family, others, community, society, nature, and the meaningful or sacred. Spirituality is expressed through beliefs, values, traditions, and practices”^(44)^. From this definition, some aspects related to Heidegger’s thought emerge, discussed in his work “Being and Time”^(14)^. The phenomenon of the search for meaning and transcendence is typical of human spirituality, and Heidegger, in his philosophy, questions the meaning of being^(14)^.

Another aspect of spirituality is the relationship of the being with itself, and Heidegger highlights that the only being that cares about its being and is capable of asking about meaning is man^(14)^. When illness arrives, it is the moment when these questions appear in consciousness, offering the possibility of living life authentically. A person with an advanced illness begins to question their own being and the meaning of life, leading to the spiritual dimension from the recognition of the existential condition of being-towards-death.

Heidegger’s existential understanding of the project of “being-there” refers to a subject that is not isolated, but together with others and in the world^(35)^. The need for relationships with others manifests itself in different ways throughout life, and at the time of illness, with the real possibility of finitude, it is necessary to share the essence of the soul’s experience^(35)^. The ontological structure is essentially relational, in the sense that “being-there” is achieved through relationships with others, and this is typical of the spiritual dimension.

Hence, recognizing finite existence allows us to live life authentically and recognize death as a possibility. On the other hand, trying to avoid the thought and experience of death, and the lack of coping with one’s own finitude, can lead to an inauthentic and alienated existence^(45)^. This avoidance approach can result in health problems and a less fulfilling life, as not coping with death can generate emotional and physical stresses, such as discomfort, pain and irritability^(30)^.

Distress is not limited to physical pain, but is something experienced in the social, psychological and existential dimensions of human beings^(46)^. Heidegger uses the concept of existential angst as a complex human experience in which all the systems that make life meaningful collapse. The absence of meaning in the world or in life is called spiritual distress^(14,29,31,44)^. To address these issues, palliative nurses must be able to provide spiritual care, with interventions focused on spiritual needs^(26,47,48)^.

Death for palliative care patients can be imminent. Death for Heidegger is unrepeatable, irreferential and insurmountable. For the philosopher, there is nothing beyond death, and he leaves God aside by stating that death leads nowhere^(14)^. The lack of full knowledge about what death and the dying process entails causes fear in many people, physical, psychological and behavioral responses, anger, depression, anguish and despair, which can have lasting effects^(49)^.

## PRACTICAL IMPLICATIONS FOR SPIRITUAL CARE IN PALLIATIVE NURSING

The question is: what does nursing care in palliative care have in common with Heidegger’s thought? As previously stated, Heidegger focused his philosophy on the study of being-there, being-in-the-world, being-with-others^(14)^. In nursing, especially in palliative care, the reason for being is to care for the “being-there” who is experiencing the phenomenon of the real proximity of death and dying.

Nursing can use the phenomenological framework to understand the experience of being in its various manifestations, as in the case of the experience lived by the “being-there” in palliative care and at the end of life. In this context, nurses can broaden their worldview and contribute to the epistemological construction based on experiences, experiences and problems. This approach covers themes related to the health of different groups, including adults, older adults, children, adolescents and women, addressing complex phenomena in their various facets^(50)^.

Heidegger proposes that the “being” of men is based on language, which is only realized through dialogue; conversation implies more than listening (physically), but it is listening. Conversation, shared language, makes the human being human^(14,31)^. People understand and perceive the meanings of what surrounds them through language. These conversations allow nursing to provide comprehensive and humanized care^(51)^. Providing comprehensive care means intentionally perceiving that dynamic private world that reveals itself as the care relationship deepens. This is more of a relationship of accompaniment, as it is built as nurses and patients to know each other and reveal themselves. In this relational process, bridges begin to be established between the two worlds: being a nurse and being a patient^(31)^, generating authentic spiritual care and empathy.

Phenomenology allows us to describe and understand the experience of these moments of encounter in healthcare among patient or family member and nurse. As we question the “being-there” about how they live a certain experience, they will enter into dialogue with us, because they have something to tell us about it and only from this sense is it possible to build a relationship of comprehensive care^(31)^.

Existential distress and spiritual distress, in the context of palliative care, can have an impact on the “being-there”, affecting the last days of life and the relationship with loved ones. It is important for nurses to reflect on the process of death and dying. They also need to recognize spiritual distress as a problem that requires understanding, care and attention, and facilitate strategies of comfort and peace for patients and their families.

Thinking about spiritual care based on Heidegger’s concepts must include some of his key ideas, especially in relation to being-towards-death (*sein-zum-tode*), care (*sorge*) and authenticity in existence^(14)^. Nursing staff can help people cope with and accept their own finitude by providing a safe space for individuals to reflect on death, process their fears, and find peace in their finitude^(28)^. Coping with death can motivate people to seek deeper meaning in their lives and find a balance between matter and spirit. Spiritual care can facilitate this search process by helping people reflect on their lives, their achievements, their relationships, and what they consider most valuable^(16,28,35,40,52)^.

Heidegger’s idea of “care” (*sorge*)^(14)^ is described as a way of being in the world that involves authentic concern for oneself and others. In spiritual care, this is possible through active and empathetic listening, by being genuinely present, and through conversations about existential themes such as the purpose of life, death, and what comes next, reflections that can help people find meaning in their lived experience^(52)^. For nurses to be genuinely present and feel confident in providing this type of care, it is essential to reflect on these philosophical and existential questions of life and death. Care, in the Heideggerian sense, is not limited to the physical, but encompasses comprehensive care. The focus on spiritual care does not imply the exclusion of other aspects of the being, but rather the recognition that the spiritual is an essential dimension of human existence, especially in moments of vulnerability, such as palliative care and the final phase of life. There is a lack of healthcare professionals’ knowledge to address this dimension in healthcare^(20)^.

The specific mention of spiritual care is justified because, during the palliative care phase, people often face deep existential questions that can cause spiritual distress. Heidegger emphasizes that the human being is a being that projects itself into the future (being-towards-death) and that the anguish in the face of death is a constitutive part of existence^(14)^. In palliative care, this existential horizon becomes more present, and it is here that spiritual care becomes a priority^(20)^.

Living authentically is another essential concept that Heidegger supports^(14)^. Spiritual care can help people live in accordance with their own values and beliefs, especially in times of crisis or at the end of life. Authenticity also involves a deep awareness of oneself and one’s place in the world^(14)^. Spiritual care can support people in self-knowledge and recognition of their own being, promoting greater clarity and inner peace. This type of care can also include meditations^(28)^, prayers, spiritual readings, or any other meaningful practice. Providing access to spiritual rituals and practices aligned with the beliefs and values of the “being-there” can facilitate a sense of connection and peace^(12,52)^.

## FINAL CONSIDERATIONS

Healthcare is complex, and approaching health from the spiritual dimension is even more so, as it forces healthcare professionals to come into contact with existential issues of the experience lived by patients and their family, and at the same time to look at themselves and their own spiritual dimension.

Another important aspect is the existential anguish experienced by nurses in palliative care settings. This phenomenon involves facing patients’ death, but also a deep reflection on their own being. Personal and professional training, together with inner self-care, emerges as a fundamental pillar for dealing with this complex reality.

Reflection based on aspects of Martin Heidegger’s existential phenomenology allows us to look at life from another perspective and become aware of the facticity of death. This philosophical basis can enrich the understanding and assistance of nursing in palliative care. It is hoped that this article will encourage nurses and other healthcare professionals to reflect on existential issues in the care of patients, families and health teams, and that these reflections can qualify care, promoting more humanized, compassionate and authentic attention in relation to those facing the end of life. Opening healthcare professionals to spirituality and spiritual care can contribute to alleviating the distress of patients, families and teams, promoting inner peace and a more authentic life.

## References

[1] Pan American Health Organization. (2019). Core Indicators 2019: Health Trends in the Americas..

[2] Abdelaal M, Avery J, Chow R, Saleem N, Fazelzad R, Mosher P (2023). Palliative care for adolescents and young adults with advanced illness: a scoping review.. Palliat Med..

[3] World Health Organization. (2020). Palliative care [Internet]..

[4] Alves RSF, Oliveira FFB. (2022). Palliative care for health professionals: advances and difficulties.. Psicol Ciênc Profissão..

[5] Radbruch L, De L, Knaul F, Wenk R, Ali Z, Bhatnaghar S (2020). Redefining palliative care: a new consensus-based definition.. J Pain Symptom Manage..

[6] Chile. Ministerio de Salud de Chile - MINSAL. Orientación técnica cuidados paliativos universales [Internet]. Chile: Departamento de Rehabilitación y Discapacidad, División de Prevención y Control de Enfermedades; 2022.

[7] Asociación Española Contra el Cáncer. Necesidades y barreras en los cuidados paliativos al final de la vida [Internet]. Madrid: Asociación Española Contra el Cáncer; 2023.

[8] Brasil. Portaria GM/MS nº 3.681, de 7 de maio de 2024. Institui a Política Nacional de Cuidados Paliativos - PNCP no âmbito do Sistema Único de Saúde - SUS, por meio da alteração da Portaria de Consolidação GM/MS nº 2, de 28 de setembro de 2017 [Internet]. Diário Oficial da União; Brasília; 22 de maio de 2024; Edição 98, Seção 1. p. 215..

[9] Guedes A, Pedrosa A, Osório M, Pedrosa T. Palliative care in pediatric oncology: perspectives of health professionals.. Rev SBPH [Internet]. 2019.

[10] Pearce MJ, Coan AD, Herndon 2nd JE, Koenig HG, Abernethy AP. (2012). Unmet spiritual care needs impact emotional and spiritual well-being in advanced cancer patients.. Support Care Cancer..

[11] Koenig HG. (2012). Religion, spirituality, and health: the research and clinical implications.. ISRN Psychiatry..

[12] Maté-Méndez J, González-Barboteo J, Calsina-Berna A, Mateo-Ortega D, Codorniu-Zamora N, Limonero-García JT (2013). The Institut Català D’Oncologia model of Palliative Care: An Integrated and Comprehensive Framework to Address the Essential needs of Patients with Advanced Cancer.. J Palliat Care..

[13] Cassell EJ. (1999). Diagnosing suffering: a perspective.. Ann Intern Med..

[14] Heidegger M. Ser y tiempo Martín Heidegger (1927) [Internet]. Traducción, prólogo y notas de Jorge Eduardo Rivera.. Chile: Escuela de Filosofía Universidad ARCIS; 1927.

[15] Inwood M. (2002). Dicionário Heidegger. Tradução de Luisa Buarque de Holanda, Revisão técnica de Marcia Sá Cavalcante Schuback..

[16] Murata H. (2003). Spiritual pain and its care in patients with terminal cancer: construction of a conceptual framework by philosophical approach.. Palliat Support Care..

[17] NANDA. 00066 Sufrimiento espiritual [Internet]. 2023 https://www.diagnosticosnanda.com/sufrimiento-espiritual/.

[18] Eshghi F, Nikfarid L, Zareiyan A. (2023). An integrative review of defining characteristic of the nursing diagnosis “spiritual distress”.. Nurs Open.

[19] Silva VA, Marcon SS, Sales CA. (2014). Percepções de familiares de pessoas portadoras de câncer sobre encontros musicais durante o tratamento antineoplásico.. Rev Bras Enferm..

[20] Balboni TA, VanderWeele TJ, Doan-Soares SD, Long KNG, Ferrell BR, Fitchett G (2022). Spirituality in Serious Illness and Health.. JAMA..

[21] PDQ® Supportive and Palliative Care Editorial Board. Spirituality in Cancer Care (PDQ®): Health Professional Version. Bethesda.

[22] Palmeira AFA, Lopes CT, Neves VR. (2023). Nursing professionals’ education on the spiritual dimension of critical patients.. Rev Gaúcha Enferm..

[23] Hamilton IJ, Morrison J, Macdonald S. (2017). Should GPs provide spiritual care?. Br J Gen Pract..

[24] Jaman-Mewes P, Oliveira MSC, Mazotti MR, Salvetti MG. (2024). Spiritual care interventions for palliative care patients: a scoping review.. Palliat Support Care..

[25] Puchalski CM, King SDW, Ferrell BR. (2018). Spiritual Considerations.. Hematol Oncol Clin North Am..

[26] Bartel M. (2004). What is spiritual? What is spiritual suffering?. J Pastoral Care Counsel..

[27] Almeida CSL, Sales CA, Marcon SS. (2014). The existence of nursing in caring for terminally ills’life: a phenomenological study.. Rev Esc Enferm USP..

[28] Fernandes MFP, Komessu JH. (2013). Desafios do enfermeiro diante da dor e do sofrimento da família de pacientes fora de possibilidades terapêuticas.. Rev Esc Enferm USP..

[29] Oliveira MFV, Carraro TE. (2011). Care in Heidegger: an ontological possibility for nursing.. Rev Bras Enferm..

[30] Sociologandoabc. El ser-para-la-muerte. La muerte es la posibilidad que habita todas mis posibilidades [Internet]. Youtube; 2013.

[31] Rivera MS, Herrera LM. (2006). Phenomenological grounds towards comprehensive nursing care.. Texto Context - Enferm..

[32] Muniz O, Berterö CM. (2007). Living with breast cancer: its effect on the life situation and the close relationship of women in Brazil.. Cancer Nurs..

[33] Sebold LF, Locks MOH, Hammerschmidt KSA, Fernandez DLR, Tristão FR, Girondi JBR. (2018). Heidegger’s hermeneutic circle: a possibility for interpreting nursing care.. Texto Context - Enferm..

[34] Seibt CL. (2018). Considerations on Heidegger’s Hermenetic Phenomenology.. Rev NUFEN..

[35] Rojas García C., Sánchez M, Giraldo M, Bossa ML, Quintero MJ, Sequea L (2022). Manual básico de enfermería paliativa [Internet].

[36] Mortari L. Filosofía del cuidado [Internet].. Concepción: Editorial Universidad del Desarrollo; 2019.

[37] Freitas GF, Merighi MAB, Fernandes MFP. The interface between phenomenology and nursing care.. Index Enferm. 2007 Oct.

[38] Hermosilla-Ávila AE, Sanhueza-Alvarado O, Chaparro-Díaz L. (2021). Palliative care and quality of life in patients with cancer during the terminal phase.. A family/patient perspective. Enferm Clin..

[39] Grøthe Å, Biong S, Grov EK. (2015). Acting with dedication and expertise: relatives’ experience of nurses’ provision of care in a palliative unit.. Palliat Support Care..

[40] Seno VL. (2010). Being-with dying: authenticity in end-of-life encounters.. Am J Hosp Palliat Care..

[41] Mendonça AVPM. Cuidados paliativos e ser-para-morte: reflexões sobre um atendimento psicológico [dissertação].. Natal: Universidade Federal do Rio Grande do Norte; 2012.

[42] Tamura K, Kikui K, Watanabe M. (2006). Caring for the spiritual pain of patients with advanced cancer: a phenomenological approach to the lived experience.. Palliat Support Care..

[43] Gonzalez LI, Aguirre A, Kraunik R, Palacios R, Sepúlveda P, Rapiman ME. (2016). The happy elderly as a being-toward-death: a phenomenological research.. Medwave..

[44] Puchalski CM, Vitillo R, Hull SK, Reller N. (2014). Improving the spiritual dimension of whole person care: reaching national and international consensus.. J Palliat Med..

[45] Hofmann L, Manfredi RA, Kaiser RM, Jafari N, Puchalski CM. (2019). Berger AM, Hinds PS, Puchalski CM, editors. Handbook of supportive oncology and palliative care: whole-person adult and pediatric care..

[46] Cassel EJ. (1982). The nature of suffering and the goals of medicine.. N Engl J Med..

[47] Ronaldson S, Hayes L, Aggar C, Green J, Carey M. (2017). Palliative care nurses’ spiritual caring interventions: a conceptual understanding.. Int J Palliat Nurs..

[48] Vincensi BB. (2019). Interconnections: spirituality, spiritual care, and patient-centered care.. Asia Pac J Oncol Nurs..

[49] Jafari H, Cheraghi MA, Pashaeypoor S, Sadat Hoseini A. (2020). Human death: a concept analysis study.. J Nurs Midwifery Sci..

[50] Amorim TV, Souza ÍEO, Salimena AMO, Padoin SMM, Melo RCJ. (2019). Operationality of concepts in Heideggerian phenomenological investigation: epistemological reflection on Nursing.. Rev Bras Enferm..

[51] Correa ML. (2016). Humanization of attention in health services: a matter of care.. Rev Cuid (Bucaramanga)..

[52] Britt KC, Acton G. (2022). Exploring the meaning of spirituality and spiritual care with help from Viktor Frankl.. J Holist Nurs..

