# Supplementary material for: Heidegger’s philosophical foundations and his contribution to palliative nursing and spiritual care
Source: Rev Esc Enferm USP. 2024 Dec 2;58:e20240155. doi: 10.1590/1980-220X-REEUSP-2024-0155en (PMC11613920; doi:10.1590/1980-220X-REEUSP-2024-0155en)
Supplement: Translation

# Translation: Fundamentos filosóficos de Heidegger e sua contribuição à enfermagem paliativa e ao cuidado espiritual*

**DOI:** 10.1590/1980-220X-REEUSP-2024-0155pt

**Authors:** Paula Jaman-Mewes, Vera Lúcia Mendes de Paula Pessoa, Lorena Campos de Souza, Marina de Góes Salvetti

**Affiliations:** 1 Universidad de los Andes, Santiago, Chile.; 2 Universidade de São Paulo, Escola de Enfermagem, São Paulo, SP, Brazil.; 3 Universidade Estadual do Ceará, Fortaleza, CE, Brazil.

**Keywords:** Enfermagem, Cuidados de Enfermagem, Cuidados Paliativos, Filosofia, Espiritualidade

## Abstract

O objetivo deste estudo foi refletir sobre o cuidado espiritual da enfermagem em cuidados paliativos sob a perspectiva conceitual de Martin Heidegger. A aproximação entre os aspectos filosóficos de Heidegger e a prática da enfermagem paliativa, com olhar clínico ao cuidado espiritual, promovem reflexões acerca da existência humana e da finitude. Estar em cuidados paliativos pode gerar angústia, perda do sentido da vida e de conexão com o mundo. A relação de cuidado entre enfermeiro e paciente, que se estabelece por meio da linguagem, favorece o cuidado integral, intencional e humanizado, e se revela à medida que essa relação de cuidado se aprofunda. Nesse processo relacional, elos começam a ser estabelecidos entre enfermeiro e paciente, podendo levar ao cuidado espiritual autêntico. Abordar a saúde a partir da dimensão espiritual é um desafio, pois obriga o profissional de saúde a entrar em contato com questões existenciais do paciente, da família e de si mesmo. Assim, a reflexão a partir de aspectos filosóficos de Heidegger nos permite tomar consciência da facticidade da morte.

## INTRODUÇÃO

A atual mudança demográfica, expressa no aumento da expectativa de vida, é um fenômeno global que vem alterando o perfil epidemiológico em diferentes contextos, com crescimento progressivo de doenças crônicas e degenerativas, como câncer, insuficiência cardíaca, doença pulmonar obstrutiva crônica, demências, entre outras^(1)^. Crianças, adolescentes e adultos jovens também enfrentam doenças ameaçadoras da vida, e assim como as pessoas de idade mais avançada, vivenciam problemas físicos, psicológicos, sociais e espirituais, que afetam não só quem os vivencia, mas todos em seu entorno^(2,3)^. O enfrentamento dessas doenças impõe questões desafiadoras para os sistemas de saúde, os profissionais de saúde e as famílias, que vivenciam os contrastes entre avanços tecnológicos e questões existenciais relacionadas à finitude, à dignidade e à qualidade da morte^(4)^.

Nota-se neste cenário a importância de um sistema de saúde eficiente, capaz de expandir a oferta de cuidados paliativos e preparar os profissionais de saúde para lidar com as demandas mais sutis de cuidado, que vão muito além do aspecto físico.

A Organização Mundial da Saúde (OMS) define os cuidados paliativos como uma abordagem que melhora a qualidade de vida de pessoas que enfrentam problemas associados a doenças potencialmente fatais e de suas famílias. Esta abordagem busca prevenir e aliviar o sofrimento por meio da identificação precoce, avaliação correta e tratamento da dor e de outros problemas, sejam eles físicos, psicossociais ou espirituais^(3,5)^. Embora os cuidados paliativos possam se intensificar na fase final de vida, devem ser oferecidos desde o diagnóstico, proporcionando apoio e acompanhamento integral à pessoa e sua família durante todo o processo de doença^(6)^.

Estudos apontam barreiras no encaminhamento dos pacientes para os cuidados paliativos, o que pode provocar atrasos no atendimento ou mesmo impedir o acesso a esses cuidados. Em casos mais extremos, o encaminhamento é adiado até alguns dias antes do falecimento ou, em muitos casos, não ocorre, devido à falta de pessoal qualificado, alta demanda de pacientes, escassez de recursos e falta de tempo. Muitas vezes, há receio e desconhecimento sobre o conceito e as implicações do cuidado paliativo, o que dificulta o atendimento adequado às necessidades dos pacientes^(7)^.

É necessário iniciar precocemente os cuidados paliativos junto ao tratamento da doença^(8)^ e abordar todas as dimensões do cuidado, incluindo corpo, mente e espírito, por meio de um cuidado autêntico que transcenda a abordagem tecnológica e curativa^(9,10,11)^. Isso deve ocorrer também durante todo o processo de morte e pós-morte, considerando a atenção oferecida no período de luto^(8)^.

Vale enfatizar que muitas vezes, no contexto de cuidados paliativos e final da vida, pacientes e familiares têm dificuldade em dar sentido ao que estão vivenciando, e muitos experimentam desesperança, solidão, medo da morte, culpa ou frustração pela incapacidade de realizar as atividades da vida diária. Além disso, a temporalidade aparece diante da consciência, e os dias e minutos de vida são limitados, gerando sofrimento, angústia e sofrimento espiritual^(12)^. O sofrimento pode ser entendido a partir de duas perspectivas: por um lado, como ameaça; e por outro lado, o sofrimento pode ocorrer quando o indivíduo sente que não possui os recursos necessários para enfrentar essa ameaça^(12,13)^. Além disso, o sofrimento pode começar com a angústia diante da possibilidade de que, se o sintoma persistir, o paciente se veja sobrecarregado ou possa perder o controle^(13)^.

A angústia é uma experiência profunda que emerge da confrontação com aspectos essenciais da existência humana, revelando a complexidade da nossa busca por significado e propósito em um mundo finito e incerto. Na filosofia existencialista, a angústia (em alemão, *angst*) é um conceito central que se relaciona à liberdade, à responsabilidade, à morte e à busca de sentido^(14,15)^. Segundo Heidegger, a angústia difere do temor por não ter um objeto específico; em contrapartida, é uma resposta à percepção da nossa própria finitude e à falta de um fundamento absoluto no mundo^(14)^. O contexto em que Heidegger descreve a angústia é considerado existencial, devido à sua ligação com a condição humana, a finitude e a autenticidade. A angústia diante da morte não é simplesmente medo de morrer; é uma compreensão profunda de que a morte é uma parte inevitável da nossa existência, o que nos permite viver de uma maneira mais autêntica^(14)^.

O sofrimento espiritual pode surgir quando as pessoas não conseguem encontrar um sentido transcendente para suas experiências ou quando sua visão de mundo e seu lugar nele são profundamente desafiados^(16,17)^. Pode surgir também quando a pessoa não está em harmonia com suas crenças e valores mais profundos. Isso pode se manifestar como um vazio interior, uma sensação de desconexão ou uma crise de fé^(18)^. A doença que ameaça a vida afeta a vida do paciente e de sua família, gerando angústia e sentimentos de vulnerabilidade, medo e incerteza, reconhecendo a necessidade de apoio para enfrentar sua condição existencial^(19)^.

O cuidado de enfermagem inclui a dimensão espiritual do ser humano, que se torna ainda mais relevante no enfrentamento de uma doença que ameaça a existência do ser. A espiritualidade é a forma como as pessoas buscam e experimentam significado, sentido e propósito na vida. Inclui um sentido de conexão e transcendência, expressando-se por meio de valores e crenças^(20)^. Segundo o Instituto Nacional do Câncer dos Estados Unidos, a espiritualidade está presente em todas as culturas, relacionando-se a sentimentos e crenças profundas, frequentemente religiosas, que incluem o sentido de paz^(21)^. Muitos profissionais, no entanto, relatam que não se sentem aptos para prestar cuidados espirituais ao paciente e sua família^(22)^.

O cuidado espiritual é “o cuidado que reconhece e responde às necessidades do espírito humano quando confrontado com traumas, doenças ou tristezas. Inclui a necessidade de significado e autoestima, a necessidade de se expressar, a necessidade de confiar na fé, ou simplesmente a necessidade de ter um ouvinte sensível”^(23)^.

Outros autores^(20)^ afirmam que o cuidado espiritual é o reconhecimento e a atenção oferecidos às pessoas em sua busca de sentido, propósito e transcendência, em momentos de doença, sofrimento ou crise. Este cuidado se concentra em reconhecer e atender às conexões profundas que a pessoa tem consigo mesma, sua família, a comunidade, a natureza e o sagrado ou significativo em sua vida. O cuidado espiritual favorece e respeita as crenças, valores, tradições e práticas espirituais do paciente, ajudando-o a encontrar paz e bem-estar integral em momentos críticos de sua vida.

Na perspectiva internacional, identificaram-se estratégias e intervenções para facilitar o cuidado espiritual em cuidados paliativos, aplicadas por diferentes profissionais de saúde, incluindo o enfermeiro, como conversas entre o paciente e um membro da equipe, intervenções de práticas religiosas, presença terapêutica, musicoterapia guiada, intervenções multidisciplinares, meditação guiada, arteterapia e intervenções combinadas com múltiplos componentes, como música, arte, terapia integrativa e reflexão^(24)^. A avaliação espiritual precoce, por meio de uma “história espiritual” como parte da história clínica integral no começo do tratamento, pode ser uma boa estratégia inicial, pois ajuda a identificar possíveis necessidades espirituais. Exemplos de instrumentos estruturados para avaliar a história espiritual são o FICA *Spiritual Assessment Tool* (F: Fé ou Crenças; I: Importância ou influência; C: Comunidade; A: Assistência) e o Questionário GES (Grupo Espiritualidade SECPAL)^(24)^.

Mas como abordar as questões espirituais do ser humano? Como enfrentar o sofrimento espiritual e existencial que surge quando uma pessoa enfrenta uma doença avançada, incurável e irreversível? São múltiplas as preocupações espirituais^(25)^, como a perda do sentido de viver, o sentimento de culpa por querer apressar a morte, a desesperança, o medo de morrer, a incerteza da finitude, o sentimento de solidão e desconexão consigo mesmo, com os outros, com o sagrado e com o meio ambiente^(26)^. Todas essas questões espirituais são aspectos que a tecnologia e os medicamentos ainda não conseguem resolver.

Questiona-se sobre a existência de uma máquina ou medicamento para tratar a desesperança. Muitas vezes, na ausência de uma solução imediata e explícita, prefere-se evitar discutir essas questões espirituais com o paciente. Contudo, é uma realidade que não pode ser ignorada. Os profissionais de saúde devem procurar formas de promover o bem-estar espiritual, aliviar o sofrimento e facilitar a morte pacífica.

O filósofo e escritor Martín Heidegger articulou fenomenologia e hermenêutica, abordagens que podem ajudar na compreensão das questões existenciais da humanidade. Sua obra “Ser e Tempo”^(14)^ é uma das mais influentes do século XX, e revela fundamentos filosóficos que podem estimular a reflexão e o diálogo entre os profissionais de saúde em diferentes contextos.

A pessoa, para Heidegger, é um “ser-aí”. O termo “ser-aí”^(14,15)^ é um conceito central na filosofia de Heidegger que se refere ao ser humano como um ente que está no mundo, imerso no seu contexto existencial concreto em tempo e espaço. Nessa perspectiva, o ser humano não é simplesmente um objeto presente no mundo como qualquer outro ente físico, mas possui uma existência única e singular. O “ser-aí” enfrenta e se envolve ativamente com questões fundamentais da sua existência, como a compreensão da sua própria finitude, a busca de sentido e autenticidade na vida, e a confrontação com a possibilidade da morte^(14)^. Outro conceito heideggeriano é “ser-no-mundo” (em alemão, *in-der-Welt-sein*), que se refere à maneira como os seres humanos existem e se relacionam com o mundo ao seu redor. Este termo destaca que nossa existência está sempre imersa em um contexto social e cultural específico. Não somos entidades isoladas, mas nossa identidade e compreensão do ser se desenvolvem em relação ao mundo que habitamos^(14)^.

Conseguir cuidar do “ser-aí” em sua totalidade, no contexto de cuidados paliativos, é um desafio para muitos profissionais, pois os confronta não apenas com a finitude da vida do ser-aí- paciente, mas também com reflexões sobre a própria finitude. Os profissionais de enfermagem paliativa precisam estar melhor preparados para lidar diariamente com as fragilidades humanas em relação à vida e à morte, pois também experimentam situações de sofrimento, angústia, medo e ansiedade^(27)^. O cuidado requer considerar a tríade paciente-família-profissional, e este ato necessita de um espaço de reflexão sobre os valores pessoais e éticos, assim como sobre o processo de morrer^(27,28)^.

Este artigo objetivou refletir sobre o cuidado espiritual da enfermagem em cuidados paliativos sob a perspectiva filosófica-conceitual de Martin Heidegger.

## ASPECTOS CONCEITUAIS DA FILOSOFIA DO SER EM HEIDEGGER

Martin Heidegger foi um dos pensadores mais importantes do século XX. Viveu na Alemanha entre 1889 e 1976. Seus escritos continuam a ser estudados até hoje e servem de base para pesquisas filosóficas em diversos campos do conhecimento, inclusive na área da saúde. O fundamento do pensamento heideggeriano é a filosofia do ser, que explora o significado e a natureza do ser humano em relação à sua existência concreta no mundo. A ontologia existencial, como Heidegger a chamou, refere-se ao estudo de como o ser humano vive, se relaciona e experimenta sua própria existência única e concreta. A reflexão sobre o ser, entendido como a essência fundamental do ser humano, constitui o ponto de partida de sua filosofia^(14)^.

A formação teológica foi o início de sua formação acadêmica. Teve sólida formação religiosa, realizou estudos humanísticos e, em 1909, iniciou a carreira eclesiástica. No entanto, após dez anos, ele se aposentou e, em 1920, foi assistente de Edmund Husserl na Universidade de Friburg. Suas raízes filosóficas são inspiradas em Heráclito, Aristóteles, Platão e Kant^(29)^. Compreender sua formação intelectual nos permite reconhecer a forma como o filósofo via o mundo.

Heidegger desenvolveu, ao longo de seus estudos, especial­mente em seu livro “Ser e Tempo”^(14)^, alguns conceitos que podem contribuir para a reflexão em relação ao ser humano que vivencia os cuidados paliativos, voltando-se para a dimensão espiritual. O arcabouço teórico do filósofo é bem vasto, mas para um adequado movimento reflexivo, optou-se pelo estabelecimento de conceitos tais como ser-aí, ser-no-mundo, ser-no-mundo com, existência (autêntica e inautêntica), angústia, ser-para-morte e cuidado, conforme descrito no Quadro 1.

**Quadro 1:** Conceitos essenciais do pensamento de Heidegger – São Paulo, SP, Brazil, 2024.

Conceitos	Definição do conceito
*Dasein* ser-aí	Heidegger concebe o ser humano como um ser que existe no mundo, um ser existencial lançado no mundo. Esta determinação aponta para uma estrutura ontológica^(14)^. O *Dasein* é concebido antecipadamente como algo que está aí, e é um ser possível (p.147). O ser-poder essencial do *Dasein* diz respeito às formas de lidar com o “mundo”, à preocupação com os outros e ao ser-poder em relação a si mesmo^(14,15)^.
Ser-no-mundo	Contém em si a relação da existência com o ser em sua totalidade. Dependendo do momento, esse conceito tem diferentes aspectos: a análise em relação ao mundo em sua mundanidade, o ser-no-mundo como co-ser e ser-si mesmo, e o ser-em, como tal^(14,15)^. O mundo tem um significado existencial, no qual ocorrem novamente diferentes possibilidades: mundo pode significar o mundo “público” de nós ou o mundo “próprio” e circundante mais próximo (doméstico)^(14)^.
Ser-no-mundo “com”	O mundo do *Dasein* é um mundo em comum. Estar dentro é co-estar com os outros^(14)^.
Existência autêntica	É o ser que assume adequadamente a sua existência, responsabilizando-o por todas as formas de perceber a sua presença no tempo^(29)^. O existente autêntico é o ser que decide por si mesmo, não se deixa influenciar pelos outros. Ele sabe que vai morrer, aceita a morte e isso não o impede de viver, não se angustia pensando na morte. O autêntico ser-aí permite ao ser viver com mais alegria^(30)^.
Existência inautêntica	É o ser que ainda nega que vai morrer. Aí ele se sobrecarrega de coisas para sufocar dentro de si a ideia de morte^(29)^. O ser inautêntico ignora a morte, e a ignorância gera angústia. A negação neurótica da morte manifesta-se na irritabilidade e em outras doenças^(30)^.
Angústia	É um estado afetivo, que permite ao ser abrir-se a uma parte da realidade. Segundo Heidegger, a angústia gera abertura ao mundo como tal, e essa realidade ultrapassa o ser-aí. É uma experiência humana e complexa em que todos os sistemas nos quais a vida encontrou sentido entram em colapso. Algo que faz com que o mundo seja percebido como sem sentido. Na angústia, o ser percebe que não tem um destino definido, não tem onde se projetar e toma consciência de que é um ser livre. A liberdade aparece e o ser pode escolher como se desenvolver^(14,15)^. Junto à liberdade vem a responsabilidade, pois cada ser percebe que as ações têm consequências. A angústia, segundo Heidegger, conecta o homem com seu ser-para a morte. O fenômeno da angústia é diferente do medo, porque emerge do próprio *Dasein*. O elemento ameaçador não se encontra em lugar nenhum, não sabemos o que é que nos angustia, o medo, que por outro lado, vem do intramundano^(14,15)^.
Ser-para-morte	Dentro de todas as possibilidades do ser-aí, Heidegger analisa a possibilidade de morrer para o ser humano. E cada ser-aí, individual e irrepetível, pergunta pelo ser e é o ser-aí que morre. O *Dasein* sabe que a morte está dentro de suas possibilidades, e este é um fenômeno individual e intransferível^(14)^.
Cuidado (*sorge*)	O cuidado é o sentido existencial do *Dasein*, do ser-aí^(14,15)^. Para Heidegger, o cuidado (*sorge*) pode ser entendido como ocupação (*besorgen*) e como preocupação (*fürsorge*), sendo intrínseco ao ser e essencialmente indivisível^(14)^. O cuidado como ocupação define um uso específico que o ser-aí faz daqueles outros seres que vêm ao seu encontro para seu uso, conferindo-lhes uma característica de utensílios ou de preocupação, explicitada na relação ou modo de ser de uma presença para com o outro, ou seja, uma espécie de relação de intersubjetividade. O cuidado como preocupação (*fürsorge*) revela-se no ser-com-o-outro^(14,15)^.

### A Fenomenologia Heideggeriana e Sua Contribuição À Enfermagem Paliativa

Para compreender o ser do homem, Heidegger utiliza o método fenomenológico, que possibilita um percurso a partir do ser-aí^(14)^. Tal método restabelece o contato com as preocupações humanas mais profundas.

Heidegger (1927) destaca que a palavra “fenomenologia” consiste em duas partes: “fenômeno” e “logos”. Ambos vêm de termos gregos. O conceito fenômeno refere-se a “aquilo que se mostra em si”, “o que é patente”, “aquilo que aparece à consciência”, e o conceito logos refere-se à “fala”, ou seja, à “palavra”. Heidegger também sustenta que o ser dos homens se baseia na linguagem e que se torna presente através do diálogo^(31)^. Como seres humanos, expressamos e compreendemos o mundo colocando em palavras as nossas experiências^(32)^. Nesse sentido, a fenomenologia refere-se a “fazer ver o que é mostrado por si mesmo”. Este é o significado formal da pesquisa fenomenológica^(14)^.

Ao percebermos algo, fazemos uso de nossos conhecimentos, experiências e posicionamentos anteriores, e a partir daí, começamos a compreender o novo movimento e a interpretar o fenômeno. Tal fato nos leva à importância de compreender a totalidade do ser, que precisa ser compreendido sem fragmentação, ou seja, o ser-aí na sua totalidade^(33)^.

A fenomenologia heideggeriana oferece-nos a possibilidade de descobrir fenômenos ocultos, como o fenômeno de ser *persona* em cuidados paliativos, porque permite entrar em contato com quem vivencia essa experiência, e, a partir dos seus discursos, podemos compreender e interpretar o que está subjacente: o fenômeno em si^(34)^.

A enfermagem paliativa atende pessoas que enfrentam doenças que afetam todo o ser no âmbito biopsicossocial e espiritual. Um desafio para a enfermagem em cuidados paliativos é compreender a dimensão espiritual, que reúne ideias filosóficas sobre a vida, seu significado e sua finalidade. A pessoa em cuidados paliativos pode experimentar sintomas espirituais, como angústia, infelicidade, descrença, desesperança, entre outros, que também deterioram significativamente a saúde^(35)^.

Essa nova circunstância na vida do indivíduo e de sua família exige cuidado integral da equipe de saúde, principalmente da equipe de enfermagem, que é quem está a maior parte do tempo junto ao paciente. Na perspectiva heideggeriana, o ato de compreender e cuidar constitui qualidade essencial de estar presente^(36,37)^. Compreender requer conhecer a condição do outro, além de ter a capacidade de sentir os sentimentos do outro^(36)^.

Compreender as experiências de vida do paciente pode ajudar a qualificar o cuidado. O método fenomenológico, na enfermagem paliativa, sugere ir às coisas elas mesmas, manifestando no compromisso com as informações compartilhadas pelos pacientes e familiares^(36)^. O enfermeiro tem a possibilidade de investigar e desvelar a situação de saúde da pessoa que está em cuidados paliativos, compreendendo a experiência vivida não só do seu ser-aí, mas do seu ser-aí-no-mundo.

Cuidar do outro envolve a intenção genuína de compreender o outro^(35)^. A compreensão da experiência vivida, por meio do método fenomenológico, oferece fundamentos para qualificar a assistência de enfermagem de forma personalizada. Na enfermagem paliativa, a aplicação dos conceitos de Heidegger, como “ser-para-a-morte”, autenticidade e compreensão da finitude, pode ser observada em diferentes estudos e na prática, abrangendo pacientes^(38)^, familiares^(38,39)^, enfermeiros^(27,40)^ e outros profissionais de saúde^(41)^.

Estudo qualitativo que investigou a experiência vivida de pacientes e enfermeiros no contexto de cuidados paliativos destacou que a interpretação cuidadosa das preocupações do paciente sobre a morte pelas enfermeiras não apenas melhora a comunicação e a compreensão, mas também facilita um diálogo existencial profundo que reflete conceitos heideggerianos, como o ser-para-a-morte, a autenticidade e a reavaliação de valores no cuidado de pacientes em final de vida^(42)^. Outra pesquisa da área da enfermagem que utilizou a fenomenologia heideggeriana demonstrou a importância de assumir a própria existência, com consciência da sua finitude e a necessidade de encontrar significado e valor na vida, mesmo nas fases mais avançadas da vida^(43)^.

## CONCEITO DE ESPIRITUALIDADE E O “SER-AÍ” EM CUIDADOS PALIATIVOS

A espiritualidade é definida como “um aspecto intrínseco e dinâmico da humanidade, através do qual as pessoas buscam o significado último, o propósito e a transcendência, e experimentam um relacionamento consigo mesmas, com a família, com os outros, com a comunidade, a sociedade, a natureza e com o significativo ou sagrado. A espiritualidade se expressa por meio de crenças, valores, tradições e práticas”^(44)^. Desta definição, emergem alguns aspectos relacionados ao pensamento de Heidegger, discutidos em sua obra “Ser e Tempo”^(14)^. O fenômeno da busca de sentido e transcendência é típico da espiritualidade do ser humano, e Heidegger, em sua filosofia, questiona sobre o sentido do ser^(14)^.

Outro aspecto da espiritualidade é a relação do ser consigo mesmo, e Heidegger destaca que o único ser que se preocupa com o seu ser e é capaz de perguntar pelo sentido é o homem^(14)^. Quando a doença chega, é o momento em que essas questões aparecem na consciência, oferecendo a possibilidade de viver a vida de forma autêntica. A pessoa com uma doença avançada começa a se questionar sobre o seu próprio ser e o sentido da vida, levando à dimensão espiritual a partir do reconhecimento da condição existencial de ser-para-morte.

A compreensão existencial de Heidegger em relação ao projeto de “ser-aí” refere-se a um sujeito que não está isolado, mas junto aos outros e no mundo^(35)^. A necessidade de relacionamento com o outro se manifesta de diferentes maneiras no decorrer da vida, e no momento da doença, com possibilidade real de finitude, é necessário compartilhar o essencial da experiência da alma^(35)^. A estrutura ontológica é essencialmente relacional, no sentido de que o “ser-aí” se faz através das relações com os outros, e isso é típico da dimensão espiritual.

Desta maneira, reconhecer a existência finita permite-nos viver a vida de forma autêntica e reconhecer a morte como uma possibilidade. Por outro lado, tentar evitar o pensamento e a experiência da morte, e a falta de confrontação com a própria finitude pode conduzir a uma existência inautêntica e alienada^(45)^. Esta abordagem de evasão pode resultar em problemas de saúde e em uma vida menos plena, já que não confrontar a morte pode gerar tensões emocionais e físicas, como desconforto, dor e irritabilidade^(30)^.

O sofrimento não se limita apenas à dor física, mas é algo experimentado nas dimensões sociais, psicológicas e existenciais do ser humano^(46)^. Heidegger utiliza o conceito de angústia existencial como uma experiência humana complexa em que todos os sistemas que fazem a vida ter sentido entram em colapso. A ausência de sentido no mundo ou na vida é chamado de sofrimento espiritual^(14,29,31,44)^. Para enfrentar essas questões, os enfermeiros paliativistas devem ser capazes de prestar cuidados espirituais, com intervenções focadas nas necessidades espirituais^(26,47,48)^.

A morte para pacientes em cuidados paliativos pode ser iminente. A morte para Heidegger é irrepetível, irreferencial e intransponível. Para o filósofo, não há nada além da morte, e ele deixa Deus de lado ao afirmar que a morte não leva a lugar nenhum^(14)^. A falta de conhecimento total sobre o que implica a morte e o processo de morrer provoca medo em muitas pessoas, respostas físicas, psicológicas e comportamentais, raiva, depressão, angústia e desespero, o que pode ter efeitos duradouros^(49)^.

## IMPLICAÇÕES PRÁTICAS PARA O CUIDADO ESPIRITUAL NA ENFERMAGEM PALIATIVA

Pergunta-se: o que a assistência de enfermagem em cuidados paliativos tem em comum com o pensamento de Heidegger? Como exposto anteriormente, Heidegger centrou a sua filosofia no estudo do ser-aí, do ser-no-mundo, do ser-com os outros^(14)^. Na enfermagem, especialmente em cuidados paliativos, a razão de ser é cuidar do “ser-aí” que está vivenciando o fenômeno da proximidade real da morte e do morrer.

A enfermagem pode utilizar o referencial fenomenológico para compreender a experiência do ser em suas diversas manifestações, como no caso da experiência vivida pelo “ser-aí” em cuidados paliativos e no final da vida. Nesse contexto, o enfermeiro pode ampliar sua visão de mundo e contribuir para a construção epistemológica baseada em vivências, experiências e problemas. Essa abordagem abrange temas voltados para a saúde de diferentes grupos, incluindo de pessoa adulta, idosa, criança, adolescente e mulher, abordando fenômenos complexos em suas diversas facetas^(50)^.

Heidegger propõe que “o ser” dos homens se baseia na linguagem, que só se concretiza através do diálogo; conversar implica mais que ouvir (fisicamente), mas é ouvir. A conversa, a linguagem compartilhada, torna humano o ser humano^(14,31)^. As pessoas entendem e percebem os significados do que as rodeia através da linguagem. Essas conversas permitem que a enfermagem preste um cuidado integral e humanizado^(51)^. Cuidar de forma integral é perceber intencionalmente aquele mundo privado dinâmico, que se revela à medida que a relação de cuidado se aprofunda. Essa é mais uma relação de acompanhamento, pois se constrói à medida que enfermeiro e paciente vão se conhecendo e se revelando. Nesse processo relacional, pontes começam a ser estabelecidas entre os dois mundos: o ser enfermeiro e o ser paciente^(31)^, gerando o cuidado espiritual autêntico e a empatia.

A fenomenologia permite descrever e compreender a experiência desses momentos de encontro na atenção em saúde entre o paciente ou familiar e o enfermeiro. À medida que questionamos o “ser-aí” sobre como ele vive determinada experiência, ele entrará em diálogo conosco, pois tem algo a nos contar sobre isso e somente a partir desse sentido é possível construir uma relação de cuidado integral^(31)^.

A angústia existencial e o sofrimento espiritual, no contexto de cuidados paliativos, podem ter impacto no “ser-aí”, afetando seus últimos dias de vida e o relacionamento com seus entes queridos. É importante que os enfermeiros reflitam sobre o processo de morte e morrer. Eles também precisam reconhecer a angústia espiritual como um problema que requer compreensão, cuidado e atenção, e facilitar estratégias de conforto e paz para os pacientes e suas famílias.

Pensar o cuidado espiritual a partir dos conceitos de Heidegger deve incluir algumas de suas ideias-chave, especialmente em relação ao ser-para-a-morte (*sein-zum-tode*), ao cuidado (*sorge*) e à autenticidade na existência^(14)^. A equipe de enfermagem pode ajudar as pessoas a confrontar e aceitar sua própria finitude, proporcionando um espaço seguro para que os indivíduos reflitam sobre a morte, processem seus medos e encontrem paz em sua finitude^(28)^. A confrontação com a morte pode motivar as pessoas a buscar um significado mais profundo em suas vidas e encontrar o equilíbrio entre a matéria e o espírito. O cuidado espiritual pode facilitar esse processo de busca, ajudando as pessoas a refletir sobre suas vidas, seus feitos, suas relações e o que consideram mais valioso^(16,28,35,40,52)^.

A ideia de “cuidado” (*sorge*) de Heidegger^(14)^ é descrita como uma maneira de ser no mundo que implica preocupação autêntica consigo mesmo e com os outros. No cuidado espiritual, isso é possível através da escuta ativa e empática, ao estar genuinamente presente, e nas conversas sobre temas existenciais, como o propósito da vida, a morte e o que vem depois, reflexões que podem ajudar as pessoas a encontrar significado na experiência vivida^(52)^. Para que os enfermeiros possam estar genuinamente presentes e se sintam seguros para oferecer este tipo de cuidado, é fundamental refletir sobre essas questões filosóficas e existenciais da vida e da morte. O cuidado, no sentido heideggeriano, não se limita ao físico, mas abrange o cuidado integral. O enfoque no cuidado espiritual não implica a exclusão dos outros aspectos do ser, mas sim o reconhecimento de que o espiritual é uma dimensão essencial da existência humana, especialmente em momentos de vulnerabilidade, como no cuidado paliativo e fase final da vida. Há carência de conhecimentos, por parte dos profissionais de saúde, para abordar essa dimensão no cuidado em saúde^(20)^.

A menção específica ao cuidado espiritual se justifica porque, durante a fase de cuidados paliativos, as pessoas geralmente enfrentam questões existenciais profundas que podem causar sofrimento espiritual. Heidegger destaca que o ser humano é um ser que se projeta para o futuro (ser-para-a-morte) e que a angústia diante da morte é uma parte constitutiva da existência^(14)^. Nos cuidados paliativos, esse horizonte existencial se torna mais presente, e é aqui que o cuidado espiritual se converte em prioridade^(20)^.

Viver de maneira autêntica é outro conceito essencial que Heidegger sustenta^(14)^. O cuidado espiritual pode ajudar as pessoas a viver de acordo com seus próprios valores e crenças, especialmente nos momentos de crise ou no final da vida. A autenticidade também implica uma profunda consciência de si mesmo e de seu lugar no mundo^(14)^. O cuidado espiritual pode apoiar as pessoas no autoconhecimento e no reconhecimento de seu próprio ser, promovendo maior clareza e paz interior. Esse tipo de cuidado também pode incluir meditações^(28)^, orações, leituras espirituais ou qualquer outra prática significativa. Oferecer acesso a rituais e práticas espirituais alinhados às crenças e valores do “ser-aí” pode facilitar um sentido de conexão e paz^(12,52)^.

## CONSIDERAÇÕES FINAIS

O cuidado em saúde é complexo, e abordar a saúde a partir da dimensão espiritual é ainda mais, pois obriga o profissional de saúde a entrar em contato com questões existenciais da experiência vivida pelo paciente e sua família, e ao mesmo tempo a olhar para si e para a sua própria dimensão espiritual.

Outro aspecto importante é a angústia existencial experimentada pelos enfermeiros em contextos de cuidados paliativos. Esse fenômeno envolve o enfrentamento da morte do paciente, mas também uma reflexão profunda sobre o seu próprio ser. A formação pessoal e profissional, juntamente com o autocuidado interior, emerge como pilar fundamental para lidar com esta realidade complexa.

A reflexão embasada em aspectos da fenomenologia existencial de Martin Heidegger permite-nos olhar a vida sob outra perspectiva e tomar consciência da facticidade da morte. Essa base filosófica pode enriquecer a compreensão e a assistência de enfermagem em cuidados paliativos. Espera-se que este artigo estimule os enfermeiros e outros profissionais de saúde a refletir sobre questões existenciais do cuidado ao paciente, à família e à equipe de saúde, e que essas reflexões possam qualificar o cuidado, promovendo uma atenção mais humanizada, compassiva e autêntica em relação àqueles que enfrentam o final da vida. A abertura dos profissionais de saúde para a espiritualidade e para o cuidado espiritual pode contribuir para aliviar o sofrimento de pacientes, famílias e equipes, promovendo paz interior e uma vida mais autêntica.
